# Prevalence of Comorbidities in Active and Reserve Service Members Pre and Post Traumatic Brain Injury, 2017-2019

**DOI:** 10.1093/milmed/usab342

**Published:** 2021-08-23

**Authors:** Tajrina Hai, Yll Agimi, Katharine Stout

**Affiliations:** Traumatic Brain Injury Center of Excellence, Silver Spring, MD 20910, USA; General Dynamics Information Technology, Falls Church, VA 22042, USA; Traumatic Brain Injury Center of Excellence, Silver Spring, MD 20910, USA; General Dynamics Information Technology, Falls Church, VA 22042, USA; Traumatic Brain Injury Center of Excellence, Silver Spring, MD 20910, USA

## Abstract

**Objective:**

To understand the prevalence of comorbidities associated with traumatic brain injury (TBI) patients among active and reserve service members in the U.S. Military.

**Methods:**

Active and reserve SMs diagnosed with an incident TBI from January 2017 to October 2019 were selected. Nineteen comorbidities associated with TBI as identified in the literature and by clinical subject matter experts were described in this article. Each patient’s medical encounters were evaluated from 6 months before to 2 years following the initial TBI diagnoses date in the Military Data Repository, if data were available. Time-to-event analyses were conducted to assess the cumulative prevalence over time of each comorbidity to the incident TBI diagnosis.

**Results:**

We identified 47,299 TBI patients, of which most were mild (88.8%), followed by moderate (10.5%), severe (0.5%), and of penetrating (0.2%) TBI severity. Two years from the initial TBI diagnoses, the top five comorbidities within our cohort were cognitive disorders (51.9%), sleep disorders (45.0%), post-traumatic stress disorder (PTSD; 36.0%), emotional disorders (22.7%), and anxiety disorders (22.6%) across severity groups. Cognitive, sleep, PTSD, and emotional disorders were the top comorbidities seen within each TBI severity group. Comorbidities increased pre-TBI to post-TBI; the more severe the TBI, the greater the prevalence of associated comorbidities.

**Conclusion:**

A large proportion of our TBI patients are afflicted with comorbidities, particularly post-TBI, indicating many have a complex profile. The military health system should continue tracking comorbidities associated with TBI within the U.S. Military and devise clinical practices that acknowledge the complexity of the TBI patient.

## INTRODUCTION

Traumatic brain injury (TBI) is a major cause of death and disability, contributing an estimated 2.87 million emergency department visits, hospitalizations, and deaths within the USA in 2014.^1^ From 2000 to 2019, over 4,00,000 active and reserve service members (SMs) in the U.S. Military have been diagnosed with TBI.^2^ Defined as being caused by a “bump, blow, or jolt to the head or a penetrating head injury that disrupts the normal function of the brain,” the severity of TBI ranges from “mild,” or a brief change in mental status or concussion to “severe,” an extended period of loss of consciousness or amnesia after the injury.^1^ The U.S. Department of Defense (DoD) considers TBI one of the “invisible wounds of war” and “one of the signature injuries of troops” returning from Afghanistan and Iraq.^3^

Several comorbidities including post-traumatic stress disorder (PTSD), cognitive disorders, visual disorders, and other conditions have been associated with TBI; the prevalence of these comorbidities vary greatly across individual patients and by TBI severity.^4–10^ Few studies have evaluated the prevalence of TBI-related comorbidities among the military population.^8,9,11–13^ Increasing our understanding of conditions co-occurring with TBI will enable clinicians to provide comprehensive patient care and improve short- and long-term health outcomes associated with TBI. This study aimed to describe the prevalence of TBI-related comorbidities among SMs.

## METHODS

### TBI Cohort

In order to be included in this study, patients must have been on active duty, or activated reserve/guard at time of injury, with their first lifetime TBI occurring between October 1, 2016, and October 30, 2019. Beneficiaries and SMs who only had a history of TBI were excluded. Because we were unable to distinguish new TBIs from repeat TBI care using electronic health records, the incident date is defined as the first hospitalization or outpatient encounter that includes a qualifying TBI diagnosis code in any diagnostic position within DoD’s medical record data. Similarly, the severity of each patient’s initial TBI diagnosis was determined using these definitions. Patients coded with multiple TBI severities during the study period were assigned the highest severity based on the DoD TBI case definition. As per DoD’s case definition practice, patients categorized as “unclassified” were recoded to the lowest TBI severity category—mild TBI.^14^

Two comparable approaches were used to identify each patient’s first lifetime TBI. For patients with their first TBI diagnosis before June 2018, we used incidence data from the Armed Forces Health Surveillance Division (AFHSD) to identify patients and their incident diagnoses date. Due to a disruption of data availability from the AFHSD at the time of the analyses, for patients with their incident TBI diagnoses date after June 2018, we used data from the Military Health System Data Repository (MDR) to identify the patient’s first TBI ambulatory encounter or hospital admission diagnoses. For this cohort, a washout period of 6 months was used to ensure patients had no prior TBI medical encounter or admission and to ensure that the TBI represented a new injury. Both approaches used the International Classification of Diseases, 10th edition, Clinical Modification (ICD-10-CM) codes based on the official DoD’s TBI case definition to identify incident TBI.^15^

### Data Sources and Medical Encounters

To identify TBI severity and selected comorbidities, we queried 10 diagnostic fields in the Comprehensive Ambulatory Patient Record and 20 diagnostic fields from the Standard Inpatient Data Record to capture ambulatory care and inpatient healthcare data in military treatment facilities. Twenty-five diagnostic code fields in the TRICARE Encounter Data-Institutional, and TRICARE Encounter Data Non-Institutional were used to capture purchased care data including ambulatory care, inpatient consultations, and the emergency department in civilian or veteran’s administration facilities. Service members with incident TBI in the deployed setting were excluded due to poor electronic healthcare record coding in the deployed setting.

Cause of injury data was not available in this study due to poor external coding in the military health system. Providers may include cause of injury information within the medical notes, which requires manual record abstraction, a process outside the scope of this study. Furthermore, only 4% of newly identified SMs with TBI are considered to be deployment-related during the study’s time period,^2^ indicating the majority of TBIs were caused by non-combat-related events.

Each patient’s medical encounters 6 months before their initial TBI diagnoses and up to 2 years post-TBI incidence were extracted from the databases if the data were available. We obtained medical encounters for the TBI cohort from October 1, 2016, through March 16, 2020.

### Comorbidities

The resulting list of comorbidities is reflective of a targeted literature review and consensus by clinical subject matter experts from the DoD TBI Center of Excellence, which among others, included a neurosurgeon, physiatrist, nurse practitioners and expert physical therapist. Traumatic brain injury Center of Excellence is a congressionally mandated collaboration of the DoD and Veterans Affairs to promote state-of-the-science care from point-of-injury to reintegration for SMs and veterans with brain injury. Within the DoD, others have studied comorbidities associated with TBI. The conditions selected were based on those studied by the AFHSD,^16^ identified by subject matter experts on TBI working in the military health system, and identified in our literature review as being associated with TBI, pre- and post-diagnoses (see supplemental references). Diagnosis code grouping was based on “parent” ICD-10-CM categories for the condition. Clinical subject matter experts in TBI reviewed and validated the conditions and corresponding codes before the analyses was conducted. Twenty-two comorbidities were identified and 19 comorbidities are shown in this article. Three comorbidities—apraxia disorders, growth hormone disorders, and psychosis—are excluded due to low prevalence within our population. Supplementary Appendix 1 lists the comorbidities and their associated ICD-10-CM codes. Post-traumatic stress disorder, emotional disorders, anxiety disorders, and depressive disorders were shown separately due to their focus in the literature among TBI patients.^4,5,11,17–19^

### Analyses

To measure the cumulative prevalence of comorbidities pre- and post-TBI, we used time-to-event analyses from a period of 180 days before patient’s initial TBI diagnoses to the first occurrence of the comorbidity. Because we examined comorbidities in relation to the incident TBI diagnosis date, with the earliest date at 6 months before the TBI diagnosis, we define the presence of these comorbidities as cumulative prevalence.

We evaluated the cumulative prevalence of each comorbidity at the following time intervals in relation to the incident TBI diagnoses: 6 months before, 3 months before, 1 month before, at the incident TBI diagnoses visit, 1 month post, 3 months post, 6 months post, 9 months post, 1 year post, and 2 years post incident TBI diagnoses. This article highlights the cumulative prevalence rates at 6 months before, 1 month before, at the incident TBI visit, and 2 years post-TBI diagnoses. The differences in cumulative prevalence by TBI severity were determined using the log-rank test. Mantel Haenszel chi-square tests and student’s *t*-tests were used to determine differences within demographic characteristics by TBI severity. SAS Software, version 9.4 (SAS Institute, Cary, NC) was used to conduct this analyses and R (R Core Team, Vienna, Austria) was used to develop the time-to-event graphs. This study was approved by the U.S. Army Public Health Review Board (DV-15-04).

## RESULTS

### Cohort Demographic Characteristics

We identified 47,299 SMs for this study. The majority of our TBI cohort had mild TBI (*n* = 42,018, 88.8%) followed by moderate (*n* = 4964, 10.5%), severe (*n* = 234, 0.5%), and penetrating TBI severity (*n* = 101, 0.2%) (Table I). Within our cohort, 81.9% were male, 64.2% were white, and 46.9% were junior enlisted officers (Supplementary Table S1). Less than half of our cohort (45.6%) were in the 18-24 age range (Table I). Most of our cohort were Army (59.9%) (Table I) and active duty (93.8%) (Supplementary Table S1). The mean patient follow-up time was 481.4 days (Supplementary Table S1). Age, sex, rank, and mean patient follow-up time differed by TBI severity and were statistically significant.

**TABLE I.** Prevalence of Comorbidities According to TBI Severity, 6 months before and up to 2 Years post-TBI Diagnoses, 2017-2019

	TBI severity	Mild *N* (%)	Moderate *N* (%)	Severe *N* (%)	Penetrating *N* (%)	All *N* (%)
Age^a^	18-24	19,352 (46.1)	2,093 (42.3)	96 (41.0)	42 (41.6)	21,583 (45.6)
	25-34	11,534 (27.5)	1,380 (27.9)	73 (31.2)	38 (37.6)	13,025 (27.5)
	35-44	8,011 (19.1)	1,030 (20.8)	44 (18.8)	15 (14.9)	9,100 (19.2)
	45-64	3,029 (7.2)	438 (8.9)	21 (9.0)	06 (5.9)	3,494 (7.4)
	Unknown	92 (0.2)	5 (0.1)	0 (0.0)	0 (0.0)	97 (0.2)
Service	Army	25,052 (59.6)	3,103 (62.7)	132 (56.4)	58 (57.4)	28,345 (59.9)
	Air Force	5,833 (13.9)	546 (11.0)	32 (13.7)	10 (9.9)	6,421 (13.6)
	Marines	5,308 (12.6)	668 (13.5)	31 (13.3)	15 (14.9)	6,022 (12.7)
	Navy	5,564 (13.2)	573 (11.6)	35 (15.0)	18 (17.8)	6,190 (13.1)
	Coast Guard/Public Health Service/Other	261 (0.6)	56 (1.1)	4 (1.7)	0 (0.0)	321 (0.7)
Comorbidities						
Top 4	Cognitive disorders	21,197 (50.4)	3,101 (62.7)	191 (81.6)	59 (58.4)	24,548 (51.9)
	Sleep disorders	18,373 (43.7)	2,725 (55.1)	138 (59.0)	48 (47.5)	21,284 (45.0)
	Post-traumatic stress disorder (PTSD)	14,895 (35.4)	1,997 (40.4)	115 (49.1)	42 (41.6)	17,049 (36.0)
	Emotional disorders	9,057 (21.6)	1,528 (30.9)	109 (46.6)	24 (23.8)	10,718 (22.7)
Other	Alcohol and substance abuse disorders	4,352 (10.4)	671 (13.6)	62 (26.5)	17 (16.8)	5,102 (10.8)
	Anxiety disorders	9,257 (22.0)	1,342 (27.1)	71 (30.3)	30 (29.7)	10,700 (22.6)
	Depressive disorders	6,478 (15.4)	865 (17.5)	58 (24.8)	21 (20.8)	7,422 (15.7)
	Ear disorders	8,263 (19.7)	1,433 (29.0)	92 (39.3)	23 (22.8)	9,811 (20.7)
	Epilepsy	394 (0.9)	120 (2.4)	20 (8.5)	7 (6.9)	541 (1.1)
	Headache	2,464 (5.9)	436 (8.8)	30 (12.8)	08 (7.9)	2,938 (6.2)
	Heterotopic disorders	16 (0.0)	7 (0.1)	2 (0.9)	1 (1.0)	26 (0.1)
	Neck disorders	1,029 (2.4)	150 (3.0)	11 (4.7)	1 (1.0)	1,191 (2.5)
	Numbness disorders	4,086 (9.7)	638 (12.9)	33 (14.1)	12 (11.9)	4,769 (10.1)
	Phobia	814 (1.9)	88 (1.8)	04 (1.7)	00 (0.0)	906 (1.9)
	Psychological and behavioral disorders	8,330 (19.8)	1,131 (22.9)	55 (23.5)	19 (18.8)	9,535 (20.2)
	Speech disorders	420 (1.0)	96 (1.9)	30 (12.8)	09 (8.9)	555 (1.2)
	Urinary disorders	1,991 (4.7)	266 (5.4)	31 (13.2)	07 (6.9)	2,295 (4.9)
	Venous embolism thrombosis (VET)	135 (0.3)	62 (1.3)	25 (10.7)	04 (4.0)	226 (0.5)
	Visual disorders	6,159 (14.7)	1,510 (30.5)	89 (38.0)	32 (31.7)	7,790 (16.5)
	Total	42,018 (100.0)	4,946 (100.0)	234 (100.0)	101 (100.0)	47,299 (100.0)

a Mantel Haenszel chi-square is significant at *P* < .001.

### Prevalence of Comorbidities

Among the 19 identified comorbidities, the five most frequent comorbidities identified during the study follow-up were cognitive disorders (51.9%), sleep disorders (45.0%), PTSD (36.0%), emotional disorders (22.7%), and anxiety disorders (22.6%). Ear disorders (20.7%), psychological behavioral disorders (20.2%), visual disorders (16.5%), depressive disorders (15.7%), and alcohol and substance abuse disorders (10.8%) rounded off the top ten conditions prevalent in our cohort throughout the study period (Table I). Cognitive disorders, sleep disorders, PTSD, and emotional disorders were the four most common conditions across TBI severities. Other comorbidities varied in frequency within each severity group. Thirty-one percentage of moderate TBI patients and 32% of penetrating TBI patients had a visual disorder diagnosis. Over 39% of severe TBI patients had an ear disorder diagnosis and over 38% had a visual disorder diagnosis.

### Time-to-Event Analyses: Top Four Comorbidities

Supplementary Appendix 2 shows the prevalence of the top four comorbidities—cognitive disorder, sleep disorder, PTSD, and emotional disorder—at different follow-up times by TBI severity.

### Cognitive Disorders

**FIGURE 1.**
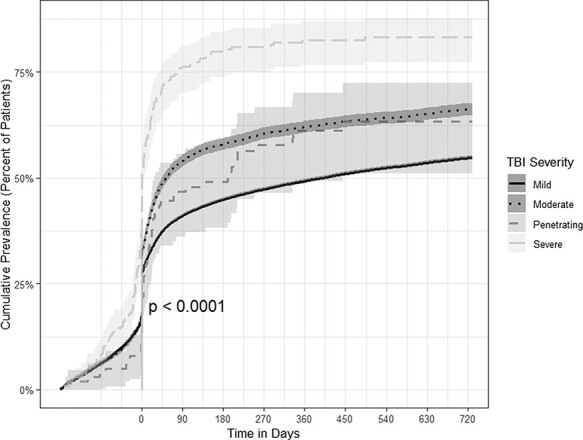
Time-to-event analyses for the prevalence of cognitive disorders by TBI severity.

Among mild TBI patients the prevalence of cognitive disorders rose from 0.1% (95% confidence interval [CI]: 0.1-0.1) 6 months before the initial TBI diagnosis to over half (54.8%; 95% CI: 54.3-55.3) by 2 years post-TBI diagnosis. Moderate TBI patients experienced a similar trend with nearly two-thirds being diagnosed with cognitive disorders (66.2%; 95% CI: 64.7-67.7) 2 years post-TBI diagnosis. The cumulative prevalence rose from 7.9% (95% CI: 4.0-15.2) 1 month before the TBI diagnoses to 83.2% (95% CI: 77.9-87.9) for severe TBI patients and from 17.9% (95% CI: 13.6-23.5) 1 month before to 63.2% (95% CI: 52.8-73.7) for penetrating TBI patients 2 years post initial diagnoses. The overall log-rank test across severities was significant (*P* < .001) (Fig. 1).

### Sleep Disorders

**FIGURE 2.**
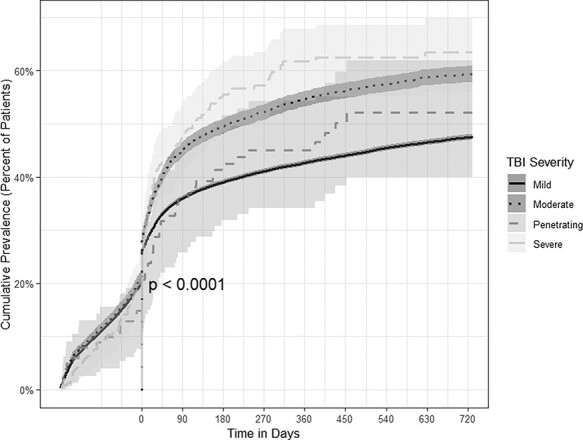
Time-to-event analyses for the prevalence of sleep disorders by TBI severity.

At the time of TBI diagnoses, about one-quarter of the mild (25.9%, 95% CI: 25.5-26.3), moderate (28.2%, 95% CI: 27.0-29.5), and severe TBI (25.2%, 95% CI: 20.1-31.3) were diagnosed with sleep disorders. Approximately, one-fifth of penetrating TBI patients (18.8%, 95% CI: 12.4-27.9) were diagnosed with sleep disorders at the time of the initial TBI diagnosis. The cumulative prevalence of sleep disorders rose across all severities. Nearly half of the mild (47.5%, 95% CI: 47.0-48.1) and penetrating TBI patients (52.1%, 95% CI: 41.8-63.3) were diagnosed with sleep disorders after 2 years. For moderate (59.3; 95% CI: 57.8-60.9) and penetrating TBI patients (63.5, 95% CI: 56.7-70.3), nearly 60% were diagnosed with sleep disorders after 2 years of follow-up (Fig. 2).

### Post-Traumatic Stress Disorder

**FIGURE 3.**
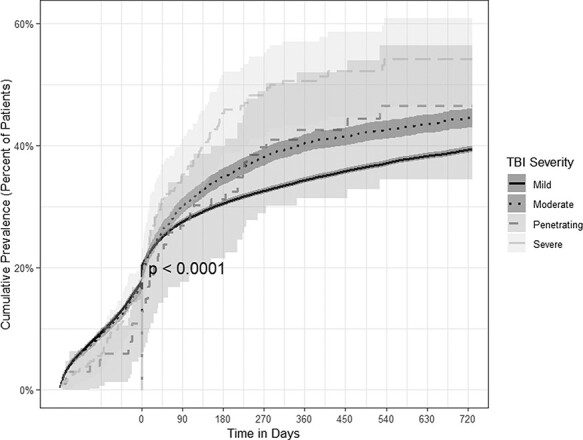
Time-to-event analyses for the prevalence of post-traumatic stress disorder (PTSD) by TBI severity.

The prevalence of PTSD across all TBI patients were nearly zero 6 months before their TBI diagnoses at the beginning of the follow-up period. Two years post diagnosis, the cumulative prevalence of PTSD rose to 39.4% (95% CI: 38.8-39.9) among mild, 44.5% (95% CI: 43.0-46.1) among moderate, and 54.1% (95% CI: 47.2-61.4) among severe TBI, and 46.5% (95% CI: 36.3-58.0) among penetrating TBI patients by 2 years post-TBI diagnosis (Fig. 3).

### Emotional Disorders

At the time of the patient’s initial TBI diagnosis, emotional disorders was around 10% among mild (9.4%; 95% CI: 9.1-9.6) and moderate (11.7%; 95% CI: 10.9-12.7) TBI patients. This prevalence rose to nearly one-quarter (24.3%; 95% CI: 23.8-24.7) among mild TBI patients and to over one-third (34.1%; 95% CI: 32.7-35.6) among moderate TBI patients 2 years after TBI diagnosis. Severe TBI patients experienced a similar trend with the cumulative prevalence rising from 16.7% (95% CI: 12.5-22.1) at the time of the TBI diagnosis to over half (50.4%; 95% CI: 43.6-57.5) 2 years post-TBI diagnosis. Among penetrating TBI patients, the cumulative prevalence rose from 5.9% (95% CI: 2.7-12.8) at the time of the TBI diagnosis to 29.6% (95% CI: 20.4-41.8) at the end of the 2-year follow-up (Supplementary Figure S1).

### Time-To-Event Analyses: Other Comorbidities

Supplementary Appendix 3 shows the prevalence of the other 15 comorbidities, at different follow-up times by TBI severity. The prevalence of alcohol or substance abuse disorders 30 days before the initial TBI diagnosis was 3.6%, 3.7%, 8.6%, and 2.0% for mild, moderate, severe, and penetrating TBI patients, respectively. At the end of the follow-up period, it rose to 11.8%, 15.1%, 28.3%, and 18.5% among mild, moderate, severe, and penetrating TBI patients, respectively.

Thirty days before their initial TBI diagnosis, 7.5% of mild, 7.0% of moderate, 9.8% of severe, and 5.9% of penetrating TBI patients had an anxiety disorder. The prevalence of anxiety disorders rose to 25.2% for mild TBI patients, 30.9% for moderate TBI patients, 34.2% for severe TBI patients, and 36.6% for penetrating TBI patients 2 years post-TBI diagnosis.

Five percentage of mild, 4.8% of moderate, 7.3% of severe, and 5.9% of penetrating TBI patients had a diagnosis of a depressive disorder 30 days before their initial TBI diagnosis. The cumulative prevalence of depressive disorders rose to 18.1% among mild TBI patients, 20.6% among moderate TBI patients, 27.2% among severe TBI patients, and 25.9% among penetrating TBI patients 2 years post-TBI diagnosis.

The prevalence of headache at the time of TBI diagnosis was 2.2% (95% CI: 2.1-2.4) among mild TBI patients, 2.1% (95% CI: 1.7-2.5) among moderate TBI patients, 3.0% (95% CI: 1.4-6.2) among severe TBI patients, and 1.0% (95% CI: 0.1-6.8) among penetrating TBI patients. Two years post-TBI diagnosis, the prevalence of a headache disorder increased to 6.5%, 9.9%, 14.1%, and 9.0% among mild, moderate, severe, and penetrating TBI patients, respectively.

## DISCUSSION

We examined a large cohort of active and reserve/guard SMs and the prevalence of TBI-related comorbidities before, during, and after their initial TBI diagnosis. Our study shows that several comorbidities afflict TBI patients before and after their incident diagnosis, which aligns with other literature.^6,18,20,21^ Cognitive disorders, sleep disorders, and PTSD have been reported to be common conditions associated with TBI.^4,12,22–25^ Cognitive impairment has been reported in TBI patients, with estimates as high as 65% among moderate and severe TBI patients,^22^ and our severe TBI population, a large cohort of SMs newly diagnosed with TBI, showed higher prevalence of cognitive conditions. Sleep disorders have been found to be a common comorbidity associated with TBI ranging from 30% to 70% of TBI patients.^23,24^ Our study found that 48% to 64% of TBI patients were diagnosed with a sleep disorder at some point during the study period, varying by TBI severity. Other studies have found that the prevalence of PTSD among TBI patients range from 27% to 44%,^4,12,25^ and our study found a higher prevalence, between 39 and 54% of TBI patients diagnosed with PTSD, varying by TBI severity. Ten percentage to 28% of TBI patients experienced emotional symptoms.^26,27^ Over one-fifth of TBI patients within our study were diagnosed with emotional disorders. Anxiety disorders, depressive disorders, and ear disorders, while lower within our cohort, fell within the range of prevalence estimates found in other research.^5,8,17,18^ Similar to findings by Gould et al., severe TBI patients had higher prevalence of comorbidities within 1 year following TBI.^20^

Headache, a commonly cited condition associated with TBI, with prevalence estimates ranging from 28% to 97%,^9,13,28,29^ was less prevalent within our cohort when compared to the literature. This may be due to a number of reasons including differences in cohort composition, time periods examined, as well as possible limitations associated with coding of headache in the military health system.^29–32^ For patients with significant polytrauma, clinicians may focus their attention on conditions of highest acuity or populate diagnostic fields with polytrauma-related diagnoses, thus not coding for all conditions experienced by the patient. In the cases of more severe brain injury, the patient’s cognitive state may limit their ability to identify and appreciate head pain.^33^ Other studies have noted higher prevalence of select comorbidities than what we found in our study; however, this may be due to smaller samples, sample composition, different study designs, and investigators looking at different comorbidities concurrently.^10,18,19,21,34^

Some conditions among the general population are prevalent before TBI, notably depression, alcohol and substance abuse, and anxiety.^6,20,34^ Our study found that less than 10% of SMs experienced these conditions before incident TBI diagnoses, although severe TBI patients had a higher prevalence of these disorders compared to other groups. Although alcohol and substance abuse is a risk factor for TBI,^35^ the condition has been underreported within the military population^7,36^ possibly due to service requirements for active duty or reluctance to discuss these issues with medical professionals.

There are several strengths to using the military health system data for this study. We were able to follow TBI patients longitudinally and assess the temporal relationship of different comorbidities to the incident TBI. Because the MDR contains patient’s electronic health record data, the data come directly from medical providers and pharmacists, resources that can generally be considered more accurate than patient self-report, particularly in longitudinal analyses.^37,38^ Additionally, because data from multiple facilities across the world are entered into the military health system, our findings are more generalizable to the military population than a single study in a single setting.^38^

## LIMITATIONS

As with any studying using electronic health records, there are a number of limitations. Surveillance bias, whereby clinicians trained specifically to look for specific comorbidities associated with TBI, may increase the prevalence of select comorbidities. This bias is particularly relevant post-TBI diagnoses as patients return for follow-up care, increasing the likelihood for other comorbidities to be identified. Because we are dealing with active duty military SMs, some comorbidities such as alcohol and substance abuse disorders and mental health disorders^37,39^ may be underreported because of the stigma associated with reporting these conditions to clinicians. Delayed reporting of comorbidities by SMs may limit the data available for this study because we restricted our analyses to 2 years after the incident TBI diagnoses.

Furthermore, the MDR is a large healthcare dataset used not only to capture diagnoses, but also healthcare utilization and billing. Due to variations in coding protocol and practices by site and by provider, conditions may be over or underrepresented in the data. The ICD-10-CM codes, the primary indicator of comorbidities used in this study, are subject to errors in coding from data entry and patients can be misclassified due to misdiagnoses or miscoding by the provider.^32,38^ Furthermore, the accuracy of ICD-10-CM diagnostic codes varies by condition.^30,32,38^ Clinicians may be frustrated from entering coding data with search queries that result in a large number of results leading to underreporting of some comorbidities. This study is limited to those conditions with their own specific ICD-10-CM codes and to the number of allowable diagnostic codes that can be entered within each data system. Although ICD-10-CM codes may underestimate specific conditions, they still serve as an effective indicator for monitoring trends.^31^ Despite these drawbacks, benefits to using the MDR outweigh its limitations for these analyses.^40^

In some studies, coding algorithms are used to verify cases using ICD-10-CM data. However, coding algorithms were not standardized for the comorbidities we selected in our study; therefore, other studies may apply slightly different coding methodologies for each comorbidity compared to our analyses.

## IMPLICATIONS FOR FUTURE RESEARCH

Injuries to the brain can have a wide variation on comorbid presentation and impact patients in the short and long term. Our research shows that a large proportion of TBI are afflicted with comorbidities, especially post-TBI, indicating many TBI patients have a complex profile, warranting a multifaceted treatment plan. We should continually monitor comorbidities among this population and extend the timeline of observation, identify comorbid clusters within this population, and determine the elevated risk associated with TBI and specific comorbidities. Monitoring comorbidities associated with TBI within the U.S. Military population is necessary to acknowledge and to improve our understanding of how these conditions impact TBI care and recovery.

## Supplementary Material

usab342_Supp
