# Supplementary material for: Prevalence of Comorbidities in Active and Reserve Service Members Pre and Post Traumatic Brain Injury, 2017-2019
Source: Mil Med. 2021 Aug 23;188(1-2):e270–7. doi: 10.1093/milmed/usab342 (PMC9825245; doi:10.1093/milmed/usab342)
Supplement: usab342_Supp [file usab342_supp.zip › MilMedicine_Appendices_30July21.docx]

# **Appendix 1. International Classification of Diseases, 10^th^ edition, Clinical Modification (ICD-10 CM) Codes Associated with select Comorbidities for Traumatic Brain Injury (TBI)**

| **Comorbidity** | **Description** | **ICD-10 CM Code** |
| --- | --- | --- |
| **Alcohol and Substance Abuse Disorders** | Alcohol | F10-F10.19, F10.2- F10.29, F10.9- F10.99 |
|  | Substance abuse | F11, F11.1 - F11.19, F11.2-F11.29 F11.9-F11.99 |
| **Anxiety Disorders** | Other anxiety disorders | F41.0-[F41.9](https://www.icd10data.com/ICD10CM/Codes/F01-F99/F40-F48/F41-/F41.9) |
| **Cognitive Disorders** | Nausea or vomiting | R11, R11.0, R11.1 – R11.2 |
|  | Fatigue | R53.1, R53.8-R53.83, G93.3 |
|  | Dizziness, vertigo | R42 |
|  | Memory loss | R41.1, R41.2, R41.3 |
|  | Other specified cognitive deficit | R41 – R41.9 |
| **Depressive Disorders** | Major Depressive Disorder, single and recurrent | F32-F32.9 |
|  | Bipolar Disorder | F31- F31.9 |
|  | Manic Disorder | F30-F30.9 |
|  | Persistent Mood Disorders | F34-F34.9 |
|  | Dysthymic Disorder | F34.1 |
| **Ear Disorders** | Conductive hearing loss | H90- H90.2, H90.A1- H90.A12 |
|  | Sensorineural hearing loss | H90.3- H90.5, H90.A2- H90.A22 |
|  | Mixed conductive and sensorineural hearing loss | H90.6-H90.8 |
|  |  | H90.A- H90.A32 |
|  | Other and unspecified hearing Loss | H91- H91.93 |
|  | Tinnitus | H93.1- H93.19 |
|  | Hyperacusis | H93.23 - H93.239 |
|  | Vestibular | H81- H81.93 |
| **Emotional Disorders** | Symptoms and signs involving emotional state including nervousness, restless, apathy, anger, hostility, and violent behavior | R45 - R45.7 |
|  | Other symptoms and signs involving emotional state | R45.8 - R45.89 |
|  | Drug induced headache | [G44.4,](https://www.icd10data.com/ICD10CM/Codes/G00-G99/G40-G47/G44-/G44.4) [G44.40,](https://www.icd10data.com/ICD10CM/Codes/G00-G99/G40-G47/G44-/G44.40)  [G44.41](https://www.icd10data.com/ICD10CM/Codes/G00-G99/G40-G47/G44-/G44.41) |
|  | Post traumatic headache | G44.319 |
| **Epilepsy** | Epilepsy/Seizures | G40- G40.919 |
| **Headache Disorders** | Tension headache | G44.209 |
|  | Cluster headache | G44.0-[G44.099](https://www.icd10data.com/ICD10CM/Codes/G00-G99/G40-G47/G44-/G44.099) |
|  | Drug induced headache | [G44.4,](https://www.icd10data.com/ICD10CM/Codes/G00-G99/G40-G47/G44-/G44.4) [G44.40,](https://www.icd10data.com/ICD10CM/Codes/G00-G99/G40-G47/G44-/G44.40)  [G44.41](https://www.icd10data.com/ICD10CM/Codes/G00-G99/G40-G47/G44-/G44.41) |
|  | Post traumatic headache | G44.319 |
| **Heterotopic Disorders** | Heterotopic ossification | M61.40, M61.50, M61.59, M61.9 |
| **Neck Disorders** | Dislocation and sprain of joints and ligaments at neck level | S13- S13.9XXS |
| **Numbness** | Numbness | R20.0-R20.9 |
| **Phobia** | Agoraphobia | F40.0- F40.02 |
|  | Social phobia | F40.1, F40.10, F40.11 |
|  | Panic disorder | F41.0 |
| **Post-traumatic stress Disorder (PTSD)** | PTSD | F43-F43.9 |
| **Psychosocial and Behavioral Disorders** | Other problems related to primary support group, including family | Z63.0- Z63.9 |
|  | Encounter for mental services for victim and perpetrator of abuse | Z69 - Z69.12, Z69.8- Z69.82 |
|  | Adult and child abuse, neglect, other maltreatment | T74.9- T74.92XS, T76.9- T76.92XS, T74- T74.01XS, T76 - T76.01XS |
|  | Housing and economic problems | Z55, Z55.9, Z59 - Z59.9 |
|  | Symptoms and signs involving emotional state | R45.8 - R45.89 |
|  | Other nonpsychotic mental disorders | F48 - F48.9 |
|  | Somatoform disorder | F45- F45.9 |
|  | War and terrorism | Z65.4, Z65.5, Z63.31 |
|  | Work employment | Z56.9 |
|  | Upbringing | Z62 – Z62.9 |
|  | Mental health | Z69 - Z69.82 |
|  | Adult and child abuse, neglect | T74 - T74.92XS |
|  | Psychosocial and Behavioral Health Problems: Suicide attempt | T14.91- T14.91XS |
| **Sleep Disorders** | Organic insomnia | G47 - G47.09, F51.03 - F51.05 |
|  | Organic hypersomnia | G47.1 - G47.19, F51.13 |
|  | Circadian rhythm sleep disorder | G47.2- G47.29 |
|  | Organic sleep apnea | G47.3 - G47.39 |
|  | Narcolepsy | G47.4 - G47.429 |
|  | Organic parasomnia | G47.5 - G47.59 |
|  | Organic sleep related movement disorders | G47.6 - G47.9 |
|  | Sleep disorders not due to a substance or known physiological condition | F51- F51.9 |
|  | Other sleep related conditions (problems related to lifestyle and sleep) | F51.12, Z72.82- Z72.9 |
| **Speech Disorders** | Aphasia | R47.01 |
|  | Dysphasia | R47.02 |
|  | Dysarthria and anarthria | R47.1 |
|  | Other speech disturbances | R47, R47.8-R47.9 |
| **Urinary Incontinence** | Urinary incontinence | N39 - N39.9, R32, N99.529 |
| **Venous Embolism and Thrombosis (VET)** | Venous embolism and thrombosis | I82 - I82.729, I82.A- I82.C29, I82.8- I82.91 |
| **Visual Disorders** | Visual Disturbances | H53.0- H53.9 |

# **Appendix 2. Select time to event data points from Kaplan-Meier survival curves for top four comorbidities: Cognitive, Sleep, Post-traumatic Stress Disorder (PTSD), and Emotional Disorders**

| **Comorbidity** | **TBI Severity** | **Time to incident TBI Diagnosis (Days)** | | | |
| --- | --- | --- | --- | --- | --- |
|  |  | **~ (-180)** | **~ (-30)** | **0** | **~730** |
|  |  | **Prevalence**  **(95% CI)** | **Prevalence**  **(95% CI)** | **Prevalence**  **(95% CI)** | **Prevalence**  **(95% CI)** |
| Cognitive Disorders | Mild | 0.1 (0.1-0.1) | 11.4 (11.1-11.7) | 27.1 (26.7-27.6) | 54.8 (54.3-55.3) |
|  | Moderate | 0.2 (0.1-0.3) | 11.1 (10.3-12.0) | 30.4 (29.2-31.8) | 66.2 (64.7-67.7) |
|  | Severe | 0.0 (0.0-0.0) | 7.9 (4.0-15.2) | 47.0 (40.9-53.6) | 83.2 (77.9-87.9) |
|  | Penetrating | 0.0 (0.0-0.0) | 17.9 (13.6-23.5) | 19.8 (13.3-29.0) | 63.2 (52.8-73.7) |
| Sleep Disorders | Mild | 0.3 (0.2-0.3) | 16.5 (16.2-16.9) | 25.9 (25.5-26.3) | 47.5 (47.0-48.1) |
|  | Moderate | 0.3 (0.2-0.6) | 17.2 (16.2-18.3) | 28.2 (27.0-29.5) | 59.3 (57.8-60.9) |
|  | Severe | 0.0 (0.0-0.0) | 15.4 (11.3-20.7) | 25.2 (20.1-31.3) | 63.5 (56.7-70.3) |
|  | Penetrating | 0.0 (0.0-0.0) | 12.9 (7.7-21.1) | 18.8 (12.4-27.9) | 52.1 (41.8-63.3) |
| Post-traumatic stress Disorder (PTSD) | Mild | 0.4 (0.4-0.5) | 14.4 (14.1-14.8) | 20.3 (19.9-20.6) | 39.4 (38.8-39.9) |
|  | Moderate | 0.3 (0.2-0.5) | 14.0 (13.1-15.0) | 18.9 (17.8-20.0) | 44.5 (43.0-46.1) |
|  | Severe | 0.4 (0.1-3.0) | 14.1 (10.2-19.3) | 18.4 (14.0-24.0) | 54.1 (47.2-61.4) |
|  | Penetrating | 0.0 (0.0-0.0) | 6.9 (3.4-14.0) | 12.9 (7.7-21.1) | 46.5 (36.3-58.0) |
| Emotional Disorders | Mild | 0.0 (0.0-0.1) | 5.0 (4.8-5.2) | 9.4 (9.1-9.6) | 24.3 (23.8-24.7) |
|  | Moderate | 0.0 (0.0-0.2) | 4.8 (4.3-5.4) | 11.7 (10.9-12.7) | 34.1 (32.7-35.6) |
|  | Severe | 0.0 (0.0-0.0) | 7.3 (4.6-11.4) | 16.7 (12.5-22.1) | 50.4 (43.6-57.5) |
|  | Penetrating | 0.0 (0.0-0.0) | 0.0 (0.0-0.0) | 5.9 (2.7-12.7) | 29.6 (20.4-41.8) |

# **Appendix 3. Select time to event data points from Kaplan-Meier survival curves for other comorbidities**

| **Comorbidity** | **TBI Severity** | **Time to Event (Days)** | | | |
| --- | --- | --- | --- | --- | --- |
|  |  | **~(-180)** | **~(-30)** | **0** | **~730** |
|  |  | **Prevalence (95% CI)** | **Prevalence**  **(95% CI)** | **Prevalence**  **(95% CI)** | **Prevalence**  **(95% CI)** |
| Alcohol and Substance Abuse Disorders | Mild | 0.2 (0.2-0.2) | 3.6 (3.4-3.8) | 5.8 (5.5-6.5) | 11.8 (11.5-12.1) |
|  | Moderate | 0.2 (0.1-0.3) | 3.7 (3.2-4.3) | 8.2 (7.4-9.6) | 15.1 (14.0-16.2) |
|  | Severe | 0.4 (0.1-3.0) | 8.5 (5.6-12.9) | 19.7 (15.1-25.4) | 28.3 (22.6-35.2) |
|  | Penetrating | 0.0 (0.0-0.0) | 2.0 (0.5-7.7) | 9.9 (5.5-17.6) | 18.5 (11.8-28.5) |
| Anxiety Disorders | Mild | 0.1 (0.1-0.2) | 7.5 (7.3-7.8) | 10.8 (10.5-11.1) | 25.2 (24.8-25.7) |
|  | Moderate | 0.0 (0.0-0.2) | 7.0 (6.3-7.7) | 10.9 (10.1-11.8) | 30.9 (29.5-32.4) |
|  | Severe | 0.0 (0.0-0.0) | 9.8 (6.6-14.4) | 13.2 (9.5-18.3) | 34.2 (27.9-41.5) |
|  | Penetrating | 0.0 (0.0-0.0) | 5.9 (2.7-12.7) | 7.9 (4.0-15.2) | 36.6 (26.2-49.4) |
| Depressive Disorders | Mild | 0.1 (0.1-0.2) | 5.0 (4.8-5.2) | 7.1 (6.9-7.4) | 18.1 (17.6-18.5) |
|  | Moderate | 0.1 (0.0-0.2) | 4.8 (4.3-5.4) | 7.1 (6.4-7.8) | 20.6 (19.3-21.9) |
|  | Severe | 0.0 (0.0-0.0) | 7.3 (4.6-11.4) | 9.4 (6.3-13.9) | 27.2 (21.6-33.9) |
|  | Penetrating | 0.0 (0.0-0.0) | 5.9 (2.7-12.7) | 7.9 (4.0-15.2) | 25.9 (17.2-38.0) |
| Ear Disorders | Mild | 0.0 (0.0-0.1) | 4.4 (4.2-4.6) | 9.5 (9.2-9.8) | 22.1 (21.6-22.5) |
|  | Moderate | 0.0 (0.0-0.2) | 5.0 (4.4-5.6) | 12.1 (11.1-13.7) | 31.7 (30.3-33.2) |
|  | Severe | 0.0 (0.0-0.0) | 4.7 (2.6-8.3) | 11.1 (7.7-15.9) | 42.2 (35.8-49.3) |
|  | Penetrating | 0.0 (0.0-0.0) | 5.1 (4.5-5.7) | 5.9 (2.7-12.7) | 23.5 (16.2-33.2) |
| Epilepsy | Mild | 0.0 (0.0-0.0) | 0.2 (0.2-0.3) | 0.4 (0.3-4.5) | 1.1 (1.0-1.2) |
|  | Moderate | 0.0 (0.0-0.1) | 0.6 (0.4-0.9) | 1.2 (0.9-1.6) | 2.6 (2.2-3.1) |
|  | Severe | 0.0 (0.0-0.0) | 0.9 (0.2-3.4) | 2.6 (1.2-5.6) | 9.4 (6.1-14.3) |
|  | Penetrating | 0.0 (0.0-0.0) | 3.0 (1.0-8.9) | 4.0 (1.5-11.2) | 6.9 (3.4-14.0) |
| Headache Disorders | Mild | 0.0 (0.0-0.0) | 0.5 (0.5-0.6) | 2.2 (2.1-2.4) | 6.5 (6.2-6.8) |
|  | Moderate | 0.0 (0.0-0.0) | 0.3 (0.2-0.6) | 2.1 (1.7-2.5) | 9.9 (9.0-10.8) |
|  | Severe | 0.0 (0.0-0.0) | 0.9 (0.2-3.4) | 3.0 (1.4-6.2) | 14.1 (9.9-19.8) |
|  | Penetrating | 0.0 (0.0-0.0) | 0.0 (0.0-0.0) | 1.0 (0.1-6.8) | 9.0 (4.6-17.5) |
| Heterotopic Disorders | Mild | 0.0 (0.0-0.0) | 0.0 (0.0-0.0) | 0.0 (0.0-0.0) | 0.1 (0.0-0.1) |
|  | Moderate | 0.0 (0.0-0.0) | 0.1 (0.0-0.2) | 0.0 (0.0-0.0) | 0.2 (0.1-0.3) |
|  | Severe | 0.0 (0.0-0.0) | 0.0 (0.0-0.0) | 0.0 (0.0-0.0) | 1.1 (0.3-4.2) |
|  | Penetrating | 0.0 (0.0-0.0) | 0.0 (0.0-0.0) | 0.0 (0.0-0.0) | 1.0 (0.1-6.8) |
| Neck Disorders | Mild | 0.0 (0.0-0.0) | 0.2 (0.2-0.3) | 1.3 (1.2-1.5) | 2.7 (2.5-2.9) |
|  | Moderate | 0.0 (0.0-0.0) | 0.3 (0.2-0.5) | 1.4 (1.1-1.8) | 3.3 (2.8-3.9) |
|  | Severe | 0.0 (0.0-0.0) | 1.7 (0.6-4.5) | 2.6 (1.2-5.6) | 3.5 (1.8-6.9) |
|  | Penetrating | 0.0 (0.0-0.0) | 0.0 (0.0-0.0) | 0.0 (0.0-0.0) | 1.1 (0.2-7.4) |
| Numbness | Mild | 0.0 (0.0-0.0) | 2.5 (2.4-2.7) | 3.8 (3.6-4.3) | 11.6 (11.2-11.9) |
|  | Moderate | 0.0 (0.0-0.0) | 3.1 (2.6-3.6) | 5.0 (4.4-5.6) | 15.1 (14.0-16.3) |
|  | Severe | 0.0 (0.0-0.0) | 3.4 (1.7-6.7) | 4.3 (2.3-7.8) | 16.5 (11.8-22.7) |
|  | Penetrating | 0.0 (0.0-0.0) | 3.0 (1.0-8.9) | 5.0 (2.1-11.5) | 13.0 (7.5-22.0) |
| Phobia | Mild | 0.0 (0.0-0.0) | 0.6 (0.5-0.7) | 0.8 (0.7-3.9) | 2.3 (2.2-2.5) |
|  | Moderate | 0.0 (0.0-0.0) | 0.4 (0.3-0.6) | 0.6 (0.3-9.8) | 2.2 (1.8-2.8) |
|  | Severe | 0.0 (0.0-0.0) | 0.4 (0.1-3.0) | 0.0 (0.0-0.0) | 2.2 (0.8-5.9) |
|  | Penetrating | 0.0 (0.0-0.0) | 0.0 (0.0-0.0) | 0.0 (0.0-0.0) | 0.0 (0.0-0.0) |
| Psychological and Behavior Disorders | Mild | 0.1 (0.1-0.1) | 6.6 (6.4-6.8) | 9.0 (8.8-9.3) | 22.9 ( 22.4-23.3) |
|  | Moderate | 0.1 (0.1-0.3) | 6.6 (5.9-7.3) | 9.0 (8.2-9.8) | 26.3 (24.9-27.7) |
|  | Severe | 0.0 (0.0-0.0) | 5.6 (3.3-9.4) | 10.3 (7.0-14.9) | 25.8 (20.3-32.4) |
|  | Penetrating | 0.0 (0.0-0.0) | 4.0 (1.5-10.2) | 7.9 (4.0-15.2) | 20.6 (13.6-30.7) |
| Speech Disorders | Mild | 0.0 (0.0-0.0) | 0.1 (0.1-0.2) | 0.3 (0.2-6.4) | 1.1 (1.0-1.3) |
|  | Moderate | 0.0 (0.0-0.0) | 0.3 (0.2-0.5) | 0.6 (0.4-6.9) | 2.1 (1.7-2.6) |
|  | Severe | 0.0 (0.0-0.0) | 1.3 (0.4-3.9) | 4.7 (2.6-8.3) | 13.6 (9.7-18.9) |
|  | Penetrating | 0.0 (0.0-0.0) | 0.0 (0.0-0.0) | 3.0 (1.0-8.9) | 10.0 (5.3-18.7) |
| Urinary Incontinence | Mild | 0.0 (0.0-0.0) | 1.4 (1.3-1.5) | 1.8 (1.7-1.9) | 5.8 (5.6-6.1) |
|  | Moderate | 0.0 (0.0-0.2) | 1.4 (1.1-1.8) | 2.0 (1.6-2.4) | 6.5 (5.7-7.3) |
|  | Severe | 0.0 (0.0-0.0) | 3.8 (2.0-7.3) | 6.8 (4.2-15.9) | 14.1 (10.0-19.6) |
|  | Penetrating | 0.0 (0.0-0.0) | 2.0 (0.5-7.7) | 3.0 (1.0-8.9) | 7.1 (3.4-14.3) |
| Venous Embolism Thrombosis (VET) | Mild | 0.0 (0.0-0.0) | 0.1 (0.1-0.2) | 0.2 (0.1-2.2) | 0.4 (0.3-0.5) |
|  | Moderate | 0.0 (0.0-0.0) | 0.3 (0.2-0.5) | 0.6 (0.4-2.9) | 1.4 (1.1-1.8) |
|  | Severe | 0.0 (0.0-0.0) | 3.4 (1.7-6.7) | 9.0 (5.9-13.4) | 10.7 (7.4-15.4) |
|  | Penetrating | 0.0 (0.0-0.0) | 0.0 (0.0-0.0) | 1.0 (0.1-6.8) | 4.0 (1.5-10.2) |
| Visual Disorders | Mild | 0.0 (0.0-0.0) | 1.5 (1.4-1.6) | 5.1 (4.9-5.3) | 16.3 (15.9-16.7) |
|  | Moderate | 0.0 (0.0-0.0) | 1.5 (1.2-1.8) | 9.5 (8.7-12.4) | 32.9 (31.5-34.4) |
|  | Severe | 0.0 (0.0-0.0) | 3.4 (1.7-6.7) | 10.2 (7.6-14.9) | 41.7 (35.2-48.9) |
|  | Penetrating | 0.0 (0.0-0.0) | 2.0 (0.5-7.7) | 5.9 (2.7-12.7) | 37.0 (27.0-49.3) |
