# Supplementary material for: Prevalence of Comorbidities in Active and Reserve Service Members Pre and Post Traumatic Brain Injury, 2017-2019
Source: Mil Med. 2021 Aug 23;188(1-2):e270–7. doi: 10.1093/milmed/usab342 (PMC9825245; doi:10.1093/milmed/usab342)
Supplement: usab342_Supp [file usab342_supp.zip › MilMedicine_SupplementalTables&Figures_22June21.docx]

**Supplemental Table 1. Baseline demographic characteristics by TBI severity and among all TBI patients, 2017-2019**

|  | **Mild** | **Moderate** | **Severe** | **Penetrating** | **All** | **df** | **p-value** |
| --- | --- | --- | --- | --- | --- | --- | --- |
| **Category** | **N=42,018 (88.8)** | **N=4,946 (10.5)** | **N=234 (0.5)** | **N=101 (0.2)** | **N=47,299 (100.0)** |  |  |
| **Sex** |  |  |  |  |  |  |  |
| Male | 34,184 (81.4) | 4,252 (86.0) | 214 (91.5) | 93 (92.1) | 38,743 (81.9) | 1 | *<.0001 |
| Female | 7,834 (18.6) | 694 (14.0) | 20 (8.6) | 8 (7.9) | 8,556 (18.1) |  |  |
| **Race** |  |  |  |  |  |  |  |
| White | 21,118 (64.1) | 2,724 (64.6) | 137 (67.8) | 67 (74.4) | 24,046 (64.2) | 1 | 0.1823 |
| Black | 5,665 (17.2) | 687 (16.3) | 24 (11.9) | 5 (5.6) | 6,381 (17.0) |  |  |
| Asian/Pacific Islander | 1,190 (3.6) | 172 (4.1) | 5 (2.5) | 1 (1.1) | 1,368 (3.7) |  |  |
| Native Americans | 213 (0.7) | 35 (0.8) | 1 (0.5) | 0 (0.0) | 249 (0.7) |  |  |
| Other | 3,318 (10.1) | 462 (11.0) | 29 (14.4) | 14 (15.6) | 3,823 (10.2) |  |  |
| Unknown | 1,457 (4.4) | 137 (3.3) | 06 (3.0) | 3 (3.3) | 1,603 (4.3) |  |  |
| **Status** |  |  |  |  |  |  |  |
| Active Duty | 39,421 (93.8) | 4,638 (93.8) | 212 (90.6) | 94 (93.1) | 44,365 (93.8) | 1 | 0.3044 |
| Reserve/Guard | 2,597 (6.2) | 308 (6.2) | 22 (9.4) | 7 (6.9) | 2,934 (6.2) |  |  |
| **Rank** |  |  |  |  |  |  |  |
| Cadet | 2,510 (6.0) | 60 (1.2) | 06 (2.6) | 00 (0.0) | 2,576 (5.5) | 1 | *<.0001 |
| Enlisted Officer, Junior (E1-E4) | 19,591 (46.6) | 2,421 (49.0) | 112 (47.9) | 51 (50.5) | 22,175 (46.9) |  |  |
| Enlisted Officer, Senior (E5-E9) | 14,571 (34.7) | 1,790 (36.2) | 86 (36.8) | 33 (32.7) | 16,480 (34.8) |  |  |
| Officer, Junior (O1-O4) | 2,410 (5.7) | 283 (5.7) | 19 (8.1) | 9 (8.9) | 2,721 (5.8) |  |  |
| Officer, Senior (O5-O10) | 2,030 (4.8) | 281 (5.7) | 9 (3.9) | 3 (3.0) | 2,323 (4.9) |  |  |
| Warrant Officer | 826 (2.0) | 101 (2.0) | 2 (0.9) | 5 (5.0) | 934 (2.0) |  |  |
| Other | 80 (0.2) | 10 (0.2) | 0 (0.0) | 0 (0.0) | 90 (0.2) |  |  |
|  | **Mean (SD)** | **Mean (SD)** | **Mean (SD)** | **Mean (SD)** | **Mean (SD)** | **T-test** |  |
| **Follow-Up Time** | 481.4 (249.9) | 481.5 (238.3) | 458.3 (239.2) | 505.2(248.2) | 481.4(248.6) | 421.0 | *<.001 |
| *Mantel Haenszel Chi-Square is significant at p<.001 | | | | | | | |

**Supplemental Figure 1. Time to Event Analyses for the Prevalence of Emotional Disorders by TBI Severity**

**
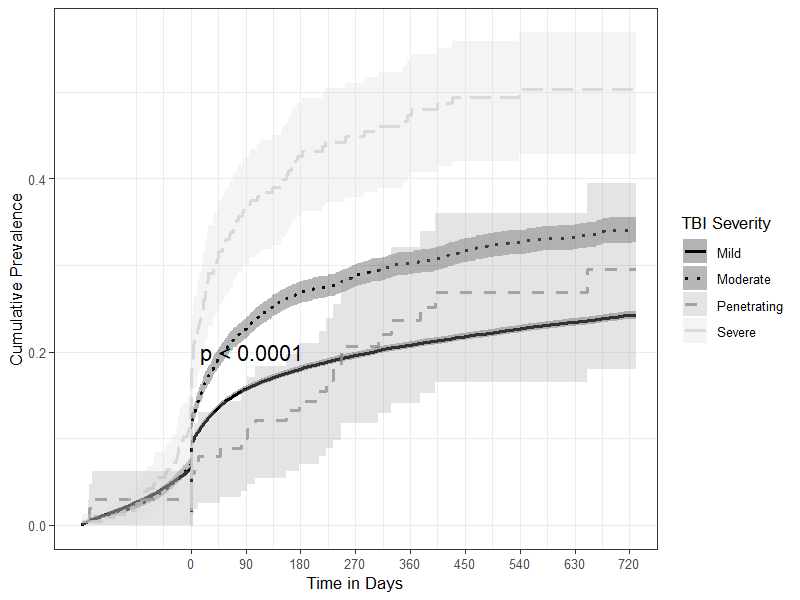
**
